# Doctors and Friendly Societies in New Zealand

**Published:** 1916-09-16

**Authors:** 


### Doctors and Friendly Societies in New Zealand.

"The Government is aware of the dispute that has arisen between the Wellington Branch of the British Medical Association and the Friendly Societies of that city, but is not proposing legislation this session to deal with the matter." This is the reply given by the Minister of Public Health to a question asked in the House of Representatives.

There has been a disagreement between the doctors and the lodges on the question of the amount of annual payment per member. For something like a quarter of a century there has existed an agreement for payment to doctors at the rate of 15s. per annum for each lodge member; and at a time when the ranks of the medical fraternity are depleted, and a scarcity of medical practitioners prevails, the doctors demanded a fresh agreement with increased terms of remuneration. They resigned their positions with the lodges, some thirty odd, and desired reappointment on a basis of 24s. instead of 15s. per member.

The lodges agreed to pay at the rate of 18s., but after several conferences between representatives from the Friendly Societies and the doctors a deadlock ensued. The lodge delegates were prepared to recommend the Societies to pay at the rate of 20s., but the doctors demanded 21s. The services of the Minister of Public Health were brought in, but his endeavours were unsuccessful in effecting a settlement. While matters were in his hands the local division of the B.M.A. forwarded certain resolutions to the lodges stating, *inter alia*, they, the doctors, could not recede from their offer at the reduced rate of 21s. per member per annum, such offer to stand open for a certain defined time; if the offer were not accepted by that date the division would decline work at a lower rate than 24s. per member per annum.

The upshot is that four fully qualified medical practitioners have been appointed from Home at a guaranteed salary of at least £700 per annum each, with payment for accouchements, operations, and administrations of anæsthetics, as well as the right of private practice. A stipulation has been made, it is said, that they shall cease to be members of the British Medical Association. And should the members of the local division refuse to consult with them it is quite probable that Parliament will introduce legislation compelling them so to do. It would be interesting to know whether any of the four are of military age, and, if so, why they are not in khaki.

